# Cardiovascular Risk Factors in a Suburban Community in Nigeria

**DOI:** 10.1155/2018/6898527

**Published:** 2018-04-01

**Authors:** Titilope Modupe Dokunmu, Omolara Faith Yakubu, Abiodun Humphrey Adebayo, Grace Iyabo Olasehinde, Shalom Nwodo Chinedu

**Affiliations:** ^1^Department of Biochemistry, Covenant University, Ota, Nigeria; ^2^Covenant University Public Health and Wellbeing Research Group (CUPHWERG), Ota, Nigeria; ^3^Department of Biological Sciences, Covenant University, Ota, Nigeria

## Abstract

The burden of hypertension, a silent killer, continues to increase in low- and middle-income countries. This study evaluated blood pressure (BP) in healthy adults to determine their risk of developing hypertension and to reduce associated morbidity of the disease. Overall, 182 subjects aged >16 years participated in the study. Systolic (SBP) and diastolic blood pressure (DBP) was measured after a resting period using mercury sphygmomanometer. Random blood glucose (RBG) concentration was also determined. Regression models were used to determine risk of high BP with *p* values < 0.05 indicating statistical difference. Prehypertension was present in 36.8% population and high BP in 31% individuals with hypertensive symptoms. DBP ≥ 90 mmHg was prevalent in the undiagnosed group, while diabetes comorbidity was detected in only 4 individuals. High BP or diabetes was not detected in those <20 year olds. Age > 35 years was an independent risk (likelihood ratio: 22.56, *p* < 0.0001); this increases to 26.48 (*p* < 0.0001) in the presence prediabetes and RBG > 100 mg/dl. Undiagnosed hypertension rate is high in the study area, and urgent interventions for large scale screening and management of the disease are required to reduce the burden of hypertension in Nigeria.

## 1. Introduction

The burden of noncommunicable diseases (NCDs) has increased in developing countries over the past decade and majority of deaths occurred in low- and middle-income countries (LMIC) [1, 2]. About 16 million people die prematurely annually from preventable NCDs, the impact of which can be lessened by reducing the risk factors associated with these diseases [3]. Diabetes and hypertension are two prevalent metabolic and leading risk factors for cardiovascular diseases, which rank the highest among the causes of mortality associated with noncommunicable diseases in Africa [3–8]. Diabetes mellitus, a chronic health condition that causes high blood sugar levels due to poor insulin production or poor utilization by the body [9], often coexists with hypertension (a systolic blood pressure ≥ 140 mmHg or diastolic blood pressure ≥ 90 mmHg), because they share common disease mechanisms; one condition exacerbates the other [10–12] although this may not always be the case [13]. The global incidence of diabetes mellitus increased in the past few decades from 108 million in 1980 to 422 million in 2014 [2] and people with uncontrolled hypertension rose from 600 million in 1980 to nearly 1 billion in 2008 [3, 4]. The highest incidence of diabetes and hypertension occurring in developing countries [2, 14, 15] has been attributed to population growth, ageing, and modernization [16].

Majority of adults (>25 years of age) are affected with these conditions and are oblivious of it; this often results in complications such as cardiovascular diseases, kidney failure, stroke, blindness, and nerve damage [2, 17–19], thus increasing the risk of deaths attributable to NCDs which is projected to rise rapidly by 2030 [3, 4, 14]. Diabetes mellitus and hypertension are often asymptomatic and undetected such that many people living with these conditions are oblivious of it [20]. Diagnosis is usually done when pathological and functional damage have progressed, thus resulting in complications and high death rates attributable to these diseases [3, 4, 6, 7, 10, 21]. Prediabetes (impaired fasting glucose and glucose tolerance) precedes the onset of diabetes, while raised blood pressure precedes hypertension; early detection and timely treatment of diabetes and hypertension are an important strategy that can be utilized in poor resource countries where NCD disease burdens are high. Studies have shown that diabetes and hypertension can be prevented or managed with drugs and lifestyle modification, thus reducing the disease burden and its sequelae [22–24]. Detection of prediabetes and prehypertension to determine persons at risk of the diseases is critical for early management and prevention of these diseases. Global prevalence of diabetes is 10% and has been on the increase in LMIC and 40% incidence of hypertension was reported in 2008 with the highest prevalence occurring in Sub-Saharan Africa; however, the prevalence varies widely across countries and regions [4, 5, 8, 25, 26]. In Nigeria, the reported prevalence of diagnosed diabetes mellitus ranges between 4.6–25% and 25–54% for hypertension, and there is a large population of undiagnosed cases [2, 11, 27–31]. In recent times, there are few studies evaluating the risks and predisposition to or early detection of hypertension and diabetes comorbidity especially in community settings in Nigeria and it has become imperative to reduce the increasing burden of cardiovascular diseases [32–34]. This study evaluates the prevalence of prehypertension and the risk of hypertension in apparently healthy adults living in semiurban communities in Nigeria where access to robust health care services is expensive and NCDs prevalence is high. The study was a cross-sectional survey which evaluated prehypertension or hypertension in healthy participants aged >16 years.

## 2. Methods

### 2.1. Study Area

The study was carried out in two suburban communities in Ota, southwest Nigeria, in April 2016 where a voluntary health survey was carried out. Ota is a populous city with a population size of over 160,000 residents. Informed consent was obtained from the participants of the study. The community leader was sought for permission and ethical approval was given by Covenant University Health Research Ethics Committee and local authority.

### 2.2. Study Population

The inclusion criteria for selecting the study participants were individuals who aged between 16 and 95 years, were nonpregnant, nondiabetic, or nonhypertensive, and were not under treatment for any of the conditions in the past two weeks and apparently healthy individuals who were willing to participate in the study. Persons with known chronic diseases and those who are ill and on antihypertensive or antidiabetic medication or aged ≤15 years were excluded from the study. Children aged <16 years (*n* = 158), pregnant women (*n* = 3), and critically ill persons (*n* = 2) were excluded from the evaluation. The power of the study was 0.90, with 7.5% margin of error and 95% confidence limit; the minimum required sample size was 171 persons. This convenient sample size of ≤200 was used for the study.

### 2.3. Study Design

The study was a cross-sectional survey, which evaluated the presence or absence of prediabetes, or diabetes mellitus and raised blood pressure (prehypertension) or hypertension onset in healthy adults.

### 2.4. Clinical Assessment

Age and demography were enquired from the participants, using a mercury sphygmomanometer; BP measurements were taken thrice, in persons with high BP; after a resting period of 15 minutes, the measurement was done again by the same field worker. Blood glucose concentration was determined in a subpopulation of participants, from blood obtained from a finger prick; random blood glucose or fasting blood glucose level was estimated using Randox® Glucometer. The attending physician clerked the individuals for further enquiries about symptoms or history of diabetes mellitus or hypertension and drug use.

### 2.5. Evaluation

Raised blood pressure or prehypertension was taken to be systolic blood pressure (SBP) of 120–139 mmHg or diastolic blood pressure (DBP) of 80–89 mmHg. Hypertension was defined as SBP ≥ 140 mmHg and/or DBP ≥ 90 mmHg [2, 35]. Prediabetes was defined as random blood glucose (RBG) of 140–199 mg/dl or fasting blood glucose of 100–125 mg/dl. Diabetes mellitus was defined as random blood glucose level of ≥200 mg/dl or fasting blood glucose of ≥126 mg/dl [6]. Others that are not within these categories were classified as normal.

### 2.6. Statistical Analysis

Data are expressed as mean ± standard deviation or proportions and were analyzed using GraphPad prism version 4 and SPSS version 16. Association between covariants and hypertension or diabetes was tested using correlation and regression models; likelihood ratio was estimated using Cox regression analysis. Chi-square test was used to compare proportions and *p* values of <0.05 were taken to indicate statistical difference.

## 3. Results

Overall, 182 individuals who met the inclusion criteria were included in the study. There were 141 (77%) females and 16 (8.7%) participants aged ≤20 years in the cohort. The mean age of the participants was 41.36 years (95% confidence interval 39.26–43.46, range 16–95 years), mean systolic blood pressure (SBP) of 119.4 mmHg (95% CI 116.3–122.5, range 90–200 mmHg), and mean diastolic blood pressure (DBP) of 78.4 mmHg (95% CI 76.4–80.3, range 50–120 mmHg).

### 3.1. Prevalence of Prehypertension and Hypertension

**Figure 1:**
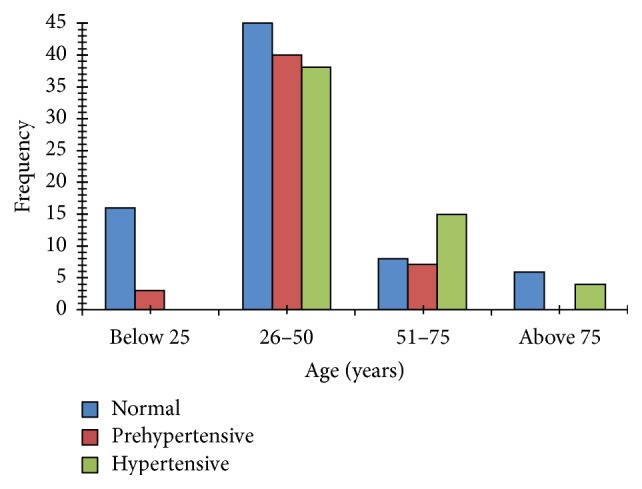
Prevalence of prehypertension and hypertension based on age in the study cohort. Prehypertension was defined as SBP 120–139 mmHg or DBP 80–90 mmHg and hypertension as SBP ≥ 140 mmHg or DBP ≥ 90 mmHg.

Prehypertension was present in 67 (36.8%) individuals from the cohort of which 72.3% were females and 3 were aged 20–23 years. Systolic blood pressure ≥ 140 mmHg was recorded in 29 (15.9%) individuals; 23 (79.3%) of them were females; SBP of ≥140 mmHg was not detected in participants <25 years old. Using standard population distribution of 50.4% females in Ota, gender adjusted prevalence of hypertension was 8.2% and 7.25% in females and males, respectively. This proportion was similar in males and females. Diastolic blood pressure ≥ 90 mmHg was present in 54 individuals (90% females). Similarly, DBP ≥ 90 mmHg was not detected in any participant <25 years. In total, 57 (31%) of the enrolled individuals had SBP ≥ 140 mmHg and/or DBP ≥ 90 mmHg, while 47% of them had both SBP ≥ 140 and DBP ≥ 90 mmHg.  Figure 1 shows the age specific prevalence of prehypertension or hypertension in the cohort.

### 3.2. Prevalence of Prediabetes and Diabetes

Blood glucose test was done in a subpopulation of participants (*n* = 79). Random blood glucose ≥ 100 mg/dl was detected in 45 participants, while prediabetes and diabetes were present in 3 and 4 individuals, respectively. None of the participants aged <20 years had prediabetes or diabetes.

### 3.3. Hypertension and Other Comorbidities

All 4 diabetic individuals also had hypertension comorbidity, while the 3 individuals with prediabetes had prehypertension. Few individuals in the cohort reported related symptoms such as headaches, weakness, and palpitations; this group is comprised mostly of adults older than 40 years. They were referred for appropriate medical follow-up.

### 3.4. Risk Assessment for Prehypertension and Hypertension

**Figure 2:**
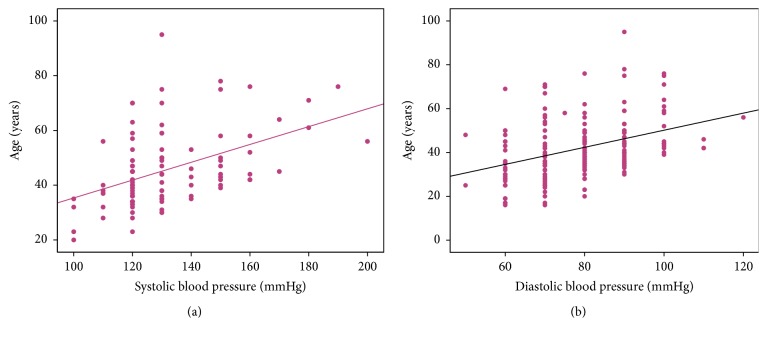
Scatter plots showing the distribution of systolic (a) (*r* = 0.48, *p* < 0.0001) and diastolic (b) blood pressure with age in the study cohort (*r* = 0.36, *p* < 0.0001).

**Figure 3:**
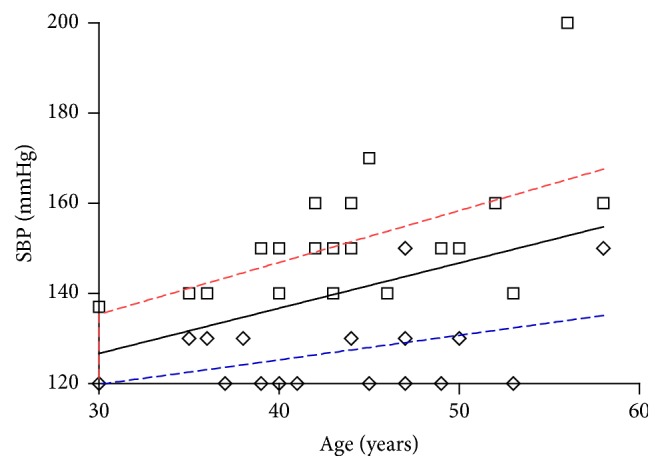
Comparison of regression plots of systolic blood pressure (SBP) and age in prehypertensive (blue dashed line), hypertensive (red dashed line), and all participants (black solid line) aged <60 years in the study cohort. Comparison of slopes: *t* = 0.235, *p* = 0.816; comparison of intercepts: *t* = 0.002, *p* = 0.999; test of coincidence, *F* = 25.365 *p* < 0.0001.

There was a significant positive correlation between prehypertension and age > 35 years (likelihood ratio 21.19, *p* < 0.0001) but not gender (likelihood ratio 0.85, *p* = 0.58) (Table 1). Likewise, there was a significant correlation between RBG and SBP (*r* = 0.22, *p* = 0.04), but not DBP (*r* = 0.18, *p* = 0.10). Prehypertension and blood sugar > 100 mg/dl showed no significant correlation (*p* = 0.06) but hypertension was significantly associated with blood sugar >100 mg/dl (likelihood ratio 6.66, *p* = 0.01). The likelihood ratio for becoming hypertensive in the individuals aged >35 years was 22.56, *p* < 0.0001. This increases to 26.48 (odds ratio 8.72, 95% CI 3.45–22.02, *p* < 0.0001) in the presence of prediabetes. Figures 2(a) and 2(b) show the plots of age and SBP (*r* = 0.48, *p* < 0.0001) or DBP (*r* = 0.36, *p* < 0.0001), respectively, and Figure 3 shows regression plots for hypertensive and prehypertensive individuals aged <70 years with similar slope and interval in the two groups.

### 3.5. Symptoms

Although the study participants appeared healthy, frequently reported symptoms experienced in the past few weeks to months include palpitations, weakness, chest pain, and headaches.

## 4. Discussion

World Health Organization identified the African region as having the highest burden of hypertension, estimated at 46% in >25 year olds [3]. Hypertension as well as diabetes mellitus contributes significantly to the burden of noncommunicable diseases globally; about three-quarters of NCD deaths occur in low- and middle-income countries [3]. Africa bears higher disease burdens due to poor preventive and expensive health schemes and genetic susceptibilities to some diseases. In our cohort, only 31.9% individuals had blood pressure within normal range, and 91.1% had blood sugar concentration within normal range. Similar proportions of male and females in this setting presented with hypertension; other studies have reported varying gender proportions in studies carried out in Nigeria [32]. A third of our population had an undiagnosed underlying condition (31.3% were hypertensive and 5% were diabetic), and a similar proportion had prehypertension or prediabetes (36.8% and 3.4%, resp.). These rates fall within range from reported studies within and outside Nigeria [26, 28, 30, 36].

Predictions that about 75% of the world hypertensive population will emerge from developing countries by 2025 [4] is imminent. With Nigeria's population of over 200 million people, indeed there is a high population with undetected emerging burden of hypertension and reports have also shown that the highest number of people with diabetes and impaired fasting glucose lives in Africa [3, 15]. This population is at increased risk for cardiovascular disease. Hypertension and diabetes comorbidity rate of 10.5% was low in this population. Our study detected only 4 individuals with prediabetes who were also hypertensive in this cohort. It would appear that comorbidity of hypertension and diabetes or the preconditions are uncommon in this setting.

Regional variation in the prevalence of diabetes and hypertension and comorbidities due to cultural and lifestyle differences, level of awareness, and socioeconomic factors such as level of income and access to affordable health care is well established. The prevalence of diabetes in our study was very low; however, it is similar to rates of 3% in eastern Nigeria [31]. For hypertension, our study reported higher rate than another study [37], where a rate of 9.7% was diagnosed in southwest Nigeria, but similar to 37.8% rate in a clinic population reported in southern [11] and eastern Nigeria [28, 34]. Our findings are not significantly disparate with reports of other studies from Nigerian population as well as other African countries [5, 25, 32, 34, 37–41]. This finding calls for routine measures and intervention to improve case detection and diagnosis of hypertension to reduce the risk of progression to complications and associated deaths.

There was a strong correlation of age with prediabetes and prehypertension. Usually, these conditions precede the disease; although not all persons with the preconditions will develop the disease, they are implicated in developing complications of the disease such as coronary heart disease and ischemic and hemorrhagic stroke [3, 19, 36]. Irrespective of lifestyle, screening and management of these diseases should be advocated in the adult age group. The likelihood for developing hypertension in prehypertensive population is high in the age group ≥35 years and consequences remain the same. NCDs caused 38 million premature deaths in 2012 and it is projected to increase to 52 million by year 2030 [3]; it is apparent that the undiagnosed population with hypertension and prehypertension of 68.1% will significantly increase soon and urgent interventions to reduce this emerging burden will help reduce morbidity and mortality caused by NCDs in LMIC. It is exigent to continue to detect the undiagnosed population and monitor trends and disease progress in affected persons for effective reduction of these public health problems.

Early detection and management of population with predisposing factors or the disease can reduce the risk of cardiovascular diseases [22–24]; however, regular screening for prehypertension or prediabetes or its knowledge as an effective prevention measure is limited across Nigeria. In a population of hypertensives, 18% with prediabetes were detected in an African study [39] and 1.1% with prediabetes in eastern Nigeria [33]. In our population, a rate of 35.7% prehypertension was recorded in the present study. Prediabetes often precedes diabetes and the risk of progression to diabetes especially in hypertensive individuals [10, 13, 39]. Undiagnosed diabetes can progress to development of complications because these conditions are often asymptomatic. The implication of undiagnosed population in Nigeria is that if not managed, they will in the next few years contribute an increase to the already high burden of morbidity and mortality associated with NCDs in LMIC [3].

The study participants generally comprised adult farmers, traders, and low income semiurban dwellers who practice more of indigenous culture, thus a presumed healthy lifestyle. Causes of these diseases are multifactorial including older age, lifestyle, diet, and underlying genetic factors, which are modifiable by environmental and other factors for the development of either hypertension or diabetes [42]. There are limitations for the reported study; questionnaires were not given to the participants to harvest lifestyle or social status that could be assessed as covariants or determinants of the observed BP and blood sugar measurements. Our study did not also evaluate lipid profiles or other biochemical determinants that predispose one to cardiovascular diseases. Previous studies have reported obesity, dyslipidemia, sedentary lifestyle, and high alcohol consumption as some important attributable risk for the development of cardiovascular diseases in Nigeria [27, 37, 43, 44]. In this population, genetic risk factors were not determined; thus, further studies are required in this area to better predict persons at higher risks of the cardiovascular diseases and preventive strategies.

The high rate of prehypertension in this population supports predictions of future increase in prevalence of NCDs in LMICs in the future [3, 4, 14, 40]. Caution is required in the interpretation of the study outcomes, gender adjusted prevalence indicates a similar prevalence of risk of cardiovascular diseases in the area; this however is contrary to other reports of a variable prevalence of hypertension based on gender [32, 43]. Our study findings buttress the fact that there is need for urgent intervention for the emerging burden of NCDs especially when the individuals are in the prehypertensive stage. Figure 3 shows a similar pattern of regression (similar slope and intercept) in hypertensive and prehypertensive individuals although the lines appear almost parallel. If no actions are taken in this population, the imminent is bound to happen.

In conclusion, our findings indicate a low prevalence of prehypertension, prediabetes, and a high undetected population with cardiovascular diseases in the study area especially in the adult population. There were no hypertensive or diabetic persons aged <25 years, suggesting that early onset of diabetes mellitus or hypertension is not common in this region. Studies have reported effective reduction in cardiovascular diseases and outcomes of treatment in persons that were managed early [23, 24, 45]. Thus, regular screening in this age group is also advocated especially in individuals with genetic susceptibility to the disease which can be detected early.

## Figures and Tables

**Table 1:** Risk assessment of prehypertension and hypertension in the cohort.

Covariants	Prehypertension odds ratio (95% CI)	Hypertension odds ratio (95% CI)	*p* value
Age (years)			
>35	4.49 (2.32–8.68)	6.34 (2.66–15.14)	<0.0001
≤35	1	1	

RBG (mg/dl)			
≥100	0.39 (0.14–1.08)^*∗*^	3.32 (1.27–8.63)	0.01
<100		1	

Gender			
Male	0.85 (0.39–1.68)	0.75 (0.36–1.57)	>0.46
Female	1		

CI: confidence interval; ^*∗*^*p* = 0.06; RBG: random blood glucose.
